# Surgical Correction of Unicoronal Craniosynostosis with Frontal Bone Symmetrization and Staggered Osteotomies

**DOI:** 10.1155/2018/3793592

**Published:** 2018-10-29

**Authors:** Seyed Esmail Hassanpour, Masoumeh Abbasnezhad, Hamidreza Alizadeh Otaghvar, Adnan Tizmaghz

**Affiliations:** ^1^Professor of Plastic Surgery, Department Of Plastic Surgery, 15 Khordad Educational Hospital, School of Medicine, Shahid Beheshti University of Medical Sciences, Tehran, Iran; ^2^Resident of Plastic Surgery, Department of Plastic Surgery, 15 Khordad Educational Hospital, School of Medicine, Shahid Beheshti University of Medical Sciences, Tehran, Iran; ^3^Associate Professor of General Surgery, Iran University of Medical Sciences, Resident of Plastic and Reconstructive Surgery, Shahid Beheshti University of Medical Sciences, Trauma & Injury Research Center, Tehran, Iran; ^4^Assistant Professor of General Surgery, Iran University of Medical Sciences, Tehran, Iran

## Abstract

**Background:**

Craniosynostosis is the premature fusion of one or more cranial sutures that produce abnormal head shape. Plagiocephaly is a general term that describes unilateral flattening of the anterior or posterior quarter of the cranium. Anterior plagiocephaly is almost always due to unilateral coronal synostosis. Early surgical treatment is the best option for these patients. The aim of this study was to investigate the surgical correction results of unicoronal craniosynostosis with frontal bone symmetrization and staggered osteotomies.

**Methods:**

All unicoronal craniosynostosis cases treated surgically from 2013 to 2016 at our hospital, with frontal bone symmetrization and staggered osteotomies and fronto-orbital advancement, were reviewed. The following variables were analyzed: sex, age, weight, hospital stay time, ICU stay time, per os (PO) starting time, anesthetic time, estimated blood loss volume (ml), estimated blood loss as percentage of total volume, surgical complication, follow-up time, and Whitaker grade. All data were analyzed with SPSS.

**Results:**

The study consisted of 33 patients (19 females, 14 males). Average age was 10.24 months, average weight was 8.97 Kg, average hospital stay time was 7.84 days, average ICU stay time was 1.69 days, average PO starting time was 1.24 days after surgery, average anesthetic time was 397.72 minutes, average estimated blood loss was 213.78 ml, and estimated blood loss as percentage of total volume was 31.69%. One case (3.03%) needed reoperation and two cases had postoperative seizure. No mortality was seen.

**Conclusion:**

It is supposed that surgical correction of unicoronal craniosynostosis with frontal bone symmetrization and staggered osteotomies results in lower blood loss, lower complication rate and reoperation, and more durable results.

## 1. Introduction

Craniosynostosis is the premature fusion of one or more cranial sutures that produce an abnormal head shape. Nonsyndromic craniosynostosis is an isolated condition without associated genetic syndromes. It occurs in approximately 1 in 1800-2500 births. In nonsyndromic forms, usually only one suture is involved and is specified as simple, but occasionally two or more sutures are involved and this condition is specified as complex [1]. Plagiocephaly is a general term denoting the unilateral flattening of the anterior or posterior quarter of the cranium. Anterior plagiocephaly is always due to unicoronal synostosis. Female to male ratio is 68%. Unicoronal synostosis produces regional growth restriction and compensatory expansion of adjacent regions and obvious fronto-orbital dysmorphology [2]. Since the 1960s and disclosure of craniofacial surgery by Tessier, different techniques for craniosynostosis have been developed, such as fronto-parietal suturectomy, lateral canthal advancement, and bilateral fronto-orbital advancement [2]. One of the problems in patients with unicoronal synostosis is frontal bone deficiency in the affected side. To resolve this problem, we use the frontal bone symmetrization method with craniotomy in the posterior aspect of the fused coronal suture. The site of craniotomy in the affected side is determined by measuring the distance in the midline, lateral, and mid-way point in the unaffected side. We used staggered osteotomies in both sides for cranial rearrangement. Fronto-orbital osteotomy was done for fronto-orbital advancement. The aim of this study is to investigate surgical correction results in 33 patients who had been treated with this method.

## 2. Patients and Method

**Figure 1:**
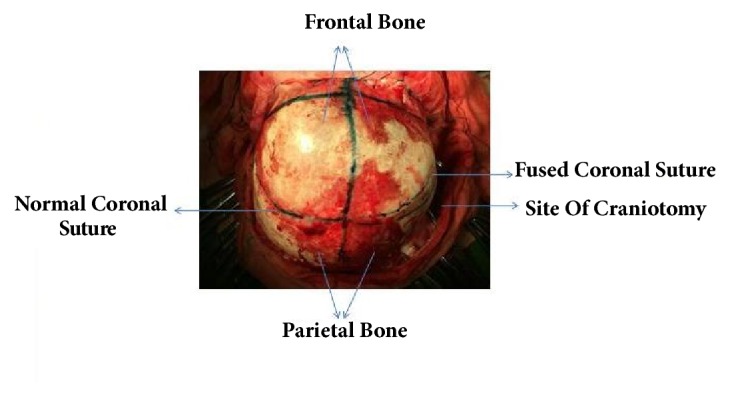
Craniotomy in unicoronal craniosynostosis was done posterior to the fused coronal suture producing bilateral frontal fragment symmetry.

**Figure 2:**
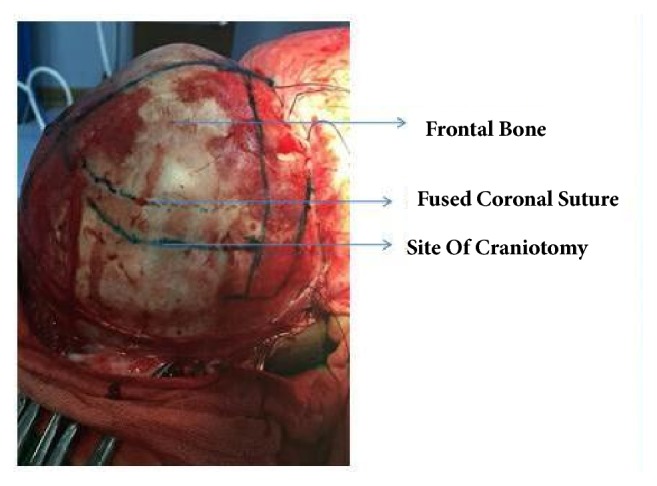
Craniotomy posterior to fused coronal suture in unicoronal craniosynostosis can add some parts of the parietal bone to the frontal bone segment.

All unicoronal craniosynostosis cases that were treated surgically from 2013 to 2016 at our hospital with frontal bone symmetrization and staggered osteotomies were reviewed. Informed consents were obtained. The following variables were analyzed: sex, age, weight, hospital stay time, ICU stay time, PO starting time, anesthetic time, estimated blood loss volume (ml), estimated blood loss as percentage of total volume, surgical complications (e.g., bleeding, infection, wound dehiscence, seizure, need for reoperation, etc.), follow-up time, and Whitaker grade. Diagnosis is based on history, physical examination, and CT-scan. Genetic testing was not carried out in our study, due to financial reasons. A team composed of a plastic and reconstructive surgeon, a neurosurgeon, a pediatrician, an ophthalmologist, and anesthesiologist visited the patient before the operation. CBC, Cr, electrolytes, and blood cross-match were checked preoperatively. All procedures were done by the same plastic and reconstructive surgeon and neurosurgeon. All patients received prophylactic antibiotics. Under GA and after skin preparation and draping, in supine position, bicorporal incision was done. The flap was elevated to the superior orbital roof with preservation of supra-orbital bundle. Frontal craniotomy was performed in both sides, but in the affected side, craniotomy was done posterior to the fused coronal suture to produce bilateral frontal fragment symmetry. The craniotomy site in the affected side is determined by measuring the distance in the midline, lateral, and mid-way points in the unaffected side. We added some parts of the parietal bone to the frontal bone segment (Figures 1 and 2).

**Figure 3:**
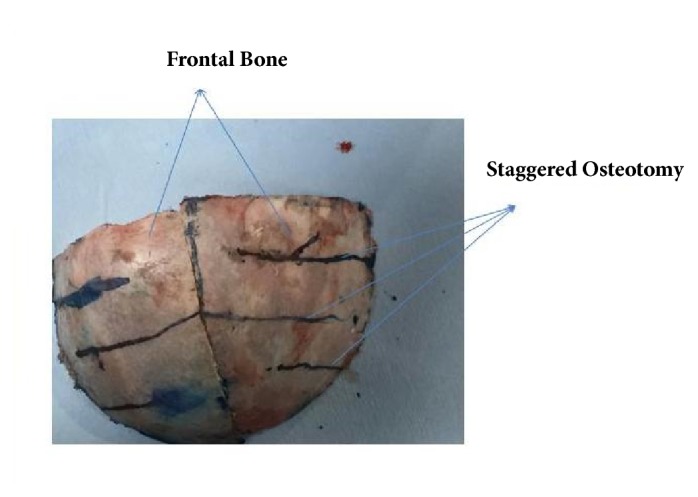
In both sides of unicoronal craniosynostosis, staggered osteotomies for remodeling and anterior cranial symmetry were done.

In both sides, staggered osteotomies for remodeling and anterior cranial symmetry were done (Figure 3).

We corrected the orbital deformity by creating the orbital bandeau, advancing it anterior to the cornea (according to the severity of deformity) and symmetrizing both orbits with fronto-orbital advancement. Postoperative management was done in a pediatric ICU as routine and then in the pediatric ward.

## 3. Results

From 2013 to 2016, 33 patients (19 females, 14 males) with unicoronal craniosynostosis were operated on in our hospital with frontal symmetrization method and staggered osteotomies. Their average age was 10.24 months (range, 4–37 months), average follow-up time was 23.42 months (range, 5–44 months), and average weight at the time of surgery was 8.97 Kg (range, 5.8–17 Kg). Average hospital stay was 7.84 days (range, 6–18 days) and average ICU stay time was 1.69 days (range, 1–5 days). Average PO starting time was 1.24 days after surgery (range, 1–5 days). Average anesthetic time was 397.72 minutes (range, 270–465 minutes). Average blood loss (intra- and postoperative from drain) that was estimated according to volume of packed cell transfused to the patient intra- and postoperative was 213.78ml (range, 60–500 ml), and average estimated blood loss as percentage of total volume was 31.69% (range 9.52-77.58%). Two cases developed postoperative seizure that was controlled with pharmacotherapy. They did not have any intracranial hemorrhage. One case (3.03%) needed reoperation 4 days postoperative, due to frontal flap dislocation and depression. No infection, wound dehiscence, or mortality was seen in our series. According to the Whitaker scale, 1 case (3.03%) was of grade IV and needed reoperation. Also, 1 case (3.03%) had forehead bony irregularity (grade III) that was proposed to be repaired, but the parents did not give consent. In total, 31 cases (93.93%) were grade I and did not need any further surgical intervention.

## 4. Discussion

Treatment goals of the craniosynostosis are adequate intracranial volume, enough for brain expansion and to minimize cognitive sequels and achieve normal cranial shape. The ideal time of surgery is controversial. Most surgeons operate on the patient as soon as possible. In nonsyndromic cases, surgery is done at around 6 months [3]. Surgical protocol involves a staged approach: (1) suture release, cranial vault decompression, and supra orbital region reshaping and advancement in infancy (6–12 months), (2) reconstructive surgery for midface abnormality in childhood (6-12 years), and (3) orthognathic surgery in adolescence (14–18 years). Exact timing and sequence of the surgical procedures are contingent upon functional and psychological aspects [3]. The categories of surgical procedure are as follows: (1) Strip craniectomy: the procedure involves cranial reshaping with fused suture removal. This method depends on the brain growth for cranial reshaping and does not treat hypoplasia or compensatory cranial changes. (2) Cranial vault remodeling technique: that is accompanied by fused suture release with direct correction of hypo plastic and compensatory cranial changes. The cranium is reshaped with different techniques including burring of the bone, rotating and reattaching the remodeled segments; bone bending, separation and barrel stave osteotomies. (3) Distraction cranioplasty: in this approach, the cranium is reshaped based on distraction osteogenesis (new bone formation) and histogenesis (new soft tissue formation) with external and internal devices. (4) Posterior release: in this method, osteotomy in the posterior cranial portion is done. This technique often is associated with distraction osteogenesis and brain expansion that induces anteroposterior diameter growth of the cranium before the fronto-orbital advancement [4]. Each technique has different results. Different craniosynostosis series are reported in the literature. G.M. Zakhary et al. reported that 100 patients were undergoing open transcranial vault reshaping with barrel-stave and orbital bandeau advancement from 1997 to 2011. Average age of the patients was 8.9 months, average weight was 9.51 Kg, and average surgical time was 216.7 minutes. Complications included 2 hematomas, 2 wound infections, 1 subgaleal abscess, 6 dural tears, 3 reoperations due to residual deformity, 4 cases requiring coronal scar revision, 1 sagittal sinus bleeding, and 1 intraoperative death [5].

Zhilin Guo et al. reviewed 165 cases over a 20-year period that were operated on with fronto-orbital advancement. Average age was 12.1 months. In 165 cases, there were 38 cases of unilateral coronal synostosis, 127 cases of bilateral deformity, and 45 cases of Crouzon syndrome. Moreover, there was one postoperative death due to intracranial bleeding and five cases of CSF leaks. In a follow-up period from 3 months to 5 years, no reoperations were needed [6].

M.P. Ferreira et al. reviewed 120 craniosynostosis cases. Average age was 7.08 months, mean surgical time was 186 minutes, and mean hospitalization time was 6.8 days. Mortality rate was 2.6%. Six patients had cardio respiratory arrest in the perioperative period, hypovolemic shock, seizure, CSF fistula, extradural hematoma, and partial wound dehiscence. Seven cases had complications such as pulmonary edema, respiratory obstruction, urinary tract infection, orbital cellulitis, and septic shock [3].

Jess-A Taylor et al. reviewed 238 nonsyndromic unicoronal craniosynostosis. These cases had undergone fronto-orbital advancement and cranial vault remodeling. They reviewed long-term aesthetic outcomes. Results showed that 55% had Whitaker class I, 6% had class II, 35% had class III, and 3% had class IV. Nasal root deviation and occipital bossing were the risk factors for Whitaker class III/IV. Bilateral cranial vault remodeling with extended unilateral bandeau had better results compared with strictly unilateral procedures. They noted traditional fronto-orbital advancement and cranial vault remodeling probably decreased the risk of intracranial hypertension, but as the patient grows, some aesthetic problems may appear [7].

Average anesthetic time in our series was 397.72 minutes. That is longer than the mentioned studies. This can be due to the time for venous and arterial line insertion, urinary catheterization, and time of intubation and extubation. We had less blood loss in our series in comparison with the mentioned studies, so less complication was seen. We had no mortality, infection, wound dehiscence, or hematoma. We had 1 case (3.03%) of reoperation due to frontal flap dislodgement. One case (3.03%) had forehead bony irregularity and was candidate for reoperation, but the parents did not give consent. Two cases (6.06%) had postoperative seizure that was controlled with pharmacotherapy.

## 5. Conclusion

It is supposed that unicoronal craniosynostosis correction with frontal bone symmetrization and staggered osteotomies can minimize bleeding, complications, and reoperation. This can be a more effective method for surgical correction of unicoronal craniosynostosis, although more studies with more cases and longer follow-up are required.

## Data Availability

The data used to support the findings of this study are available from the corresponding author upon request.
